# Y-23 mediated genetic data analysis of endogamous Brahmin population of Rajasthan, India

**DOI:** 10.1016/j.dib.2022.108061

**Published:** 2022-03-16

**Authors:** Shivkant Sharma, Ritu Yadav, Vivek Sahajpal, Mugdha Singh, Shalu Ranga, Lokesh Kadian, Chetna Yadav, Ankush Patial, Nisha Devi, Parul Ahuja

**Affiliations:** a Department of Genetics, Maharshi Dayanand University, Rohtak, 124001, India; b DNA Division, Directorate of Forensic Services, Himachal Pradesh 171218, India; c Laboratory of Genomics and Profiling Applications, Centre for DNA Fingerprinting and Diagnostics, Uppal, Hyderabad, Telangana, India

**Keywords:** Population genetics, PowerPlex® Y-23, Rajasthan, Brahmin, STRAF

## Abstract

India's largest state Rajasthan is known for its variable population groups including castes, communities and tribes. In the present article, Y-STR polymorphisms of hundred unrelated healthy male volunteers from the Brahmin population of Rajasthan, India were investigated using the Powerplex® Y-23 PCR amplification kit. Total 94 distinct haplotypes were obtained out of them 93 were singletons. Haplotype Diversity (HD) and Discrimination Capacity (DC) for the population were 0.644 and 0.9894 respectively. The Intra-population relationship between the present population data and other reported Indian populations was examined through Multidimensional Scaling (MDS) Plot, which shows the Brahmin population of Rajasthan lies in a cluster with the Brahmin populations of Haryana and Maharashtra. Data generated with 23 Y-STR markers is submitted on Y chromosome haplotype reference database (YHRD) (yhrd.org) and it will robust the forensic database of the Rajasthan population of India.

## Specifications Table

**Table:** 

Subject	*Genetics*
Specific subject area	*Forensic Genetics, DNA Profiling, Population Genetics*
Type of data	Supplementary tables (7) and figures (3)
How the data were acquired	Blood Samples on FTA cards were purified with lab modified method. The reaction mixture was then subjected to PCR amplification using PowerPlex® Y-23 PCR amplification kit (Promega, USA) followed by capillary electrophoresis. Analysis of the raw data was done with GenAlEx 6.5 and STRAF Software.
Data format	Raw and Analysed
Description of data collection	Genetic data of 100 unrelated healthy Brahmin male from Rajasthan was generated using PowerPlex® Y-23 PCR amplification kit. Y-STR alleles for each locus were called using GeneMapper ID v3.2 software and compared with standard allelic ladder. WEN ILS 500 was used as an internal lane standard. Raw STR data were then compiled in table using Microsoft Office Excel 2010.
Data source location	Rajasthan, India (Longitude 27.0238° N and Latitude 74.2179° E)
Data accessibility	Repository name: YHRD Data identification number: YC000476 Direct URL to data: https://yhrd.org/YC000476 All data are available with the article as supplementary files.

## Value of the Data

• Generated 23 Y-STR data can be used as reference for the estimation of genetic relatedness among various Indian populations and with the other populations worldwide.
• This population data may provide an important source of information for the investigations of forensic relevance in forensic laboratories.
• The data strengthens the Y-STR Haplotype Reference Database (YHRD) of Indian population that could be conducive to anthropology, population origin, evolution, and other related researches in future.
• The raw Y-STR data of Rajasthan Brahmin can be re-analysed for validation and comparison purpose.

## 1 Data Description

**Fig. 1:**
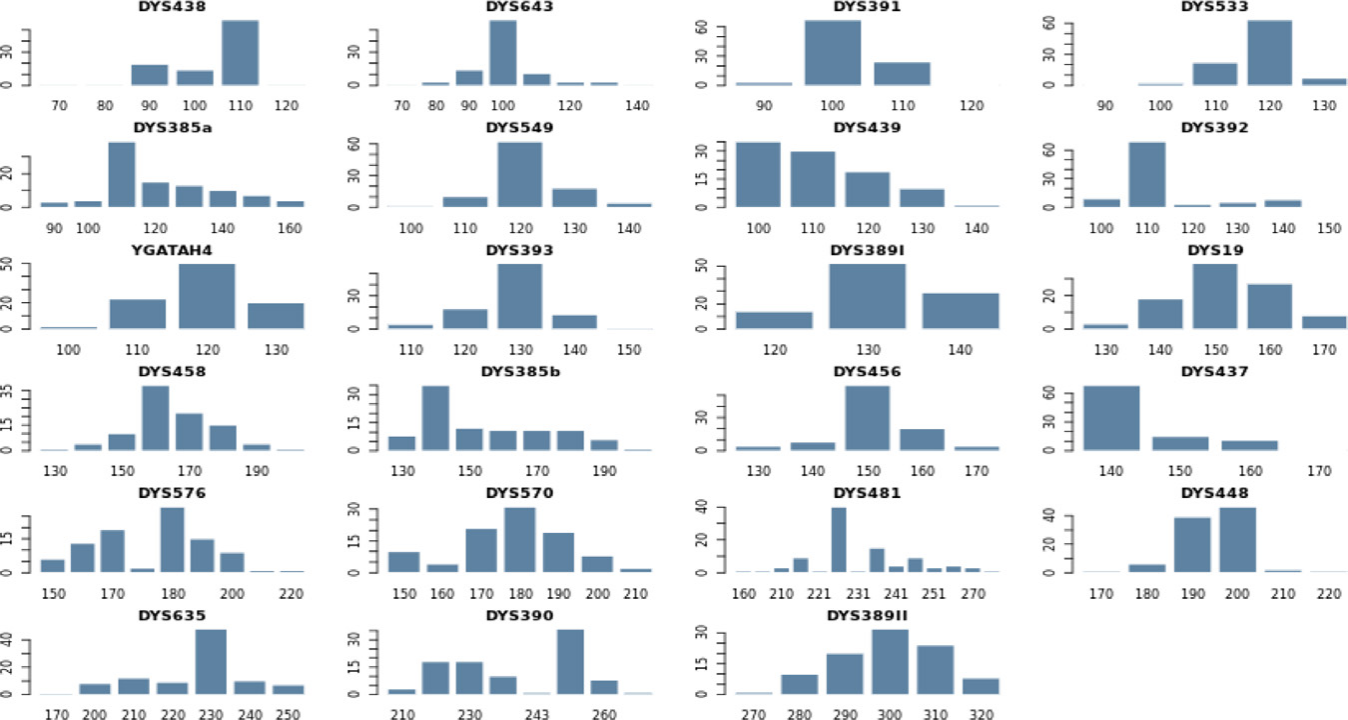
Distribution of allelic frequencies per locus on 23 Y-STR loci in Rajasthan Brahmin population using STRAF software.

In this article, we provide Y-STR data for the endogamous Brahmin population of Rajasthan, India. Total ninety-five complete haplotypes were generated using PowerPlex® Y-23 PCR amplification kit. Minimal, Powerplex Y and Y Filer marker systems were also used for evaluation (Supplementary Table 1). Due to mutations originating in the Y-homologue of the amelogenin gene, one individual was reported with the amelogenin Y-deletion, which was further confirmed by the autosomal STRs DNA profiling. Four partial profiles were obtained which were excluded from the analysis. Total 140 different alleles were found with an average value of 6.087 per locus. Overall, 26 different alleles were found ranging from 7 to 32. Maximum number of different alleles (10) was found on locus DYS481 and minimum number of different alleles (3) was found on locus DYS389I. Allelic frequency of the Rajasthan Brahmin population is enlisted in Supplementary Table 2 and allele frequencies per locus of this population are given in Fig. 1.

**Fig. 2:**
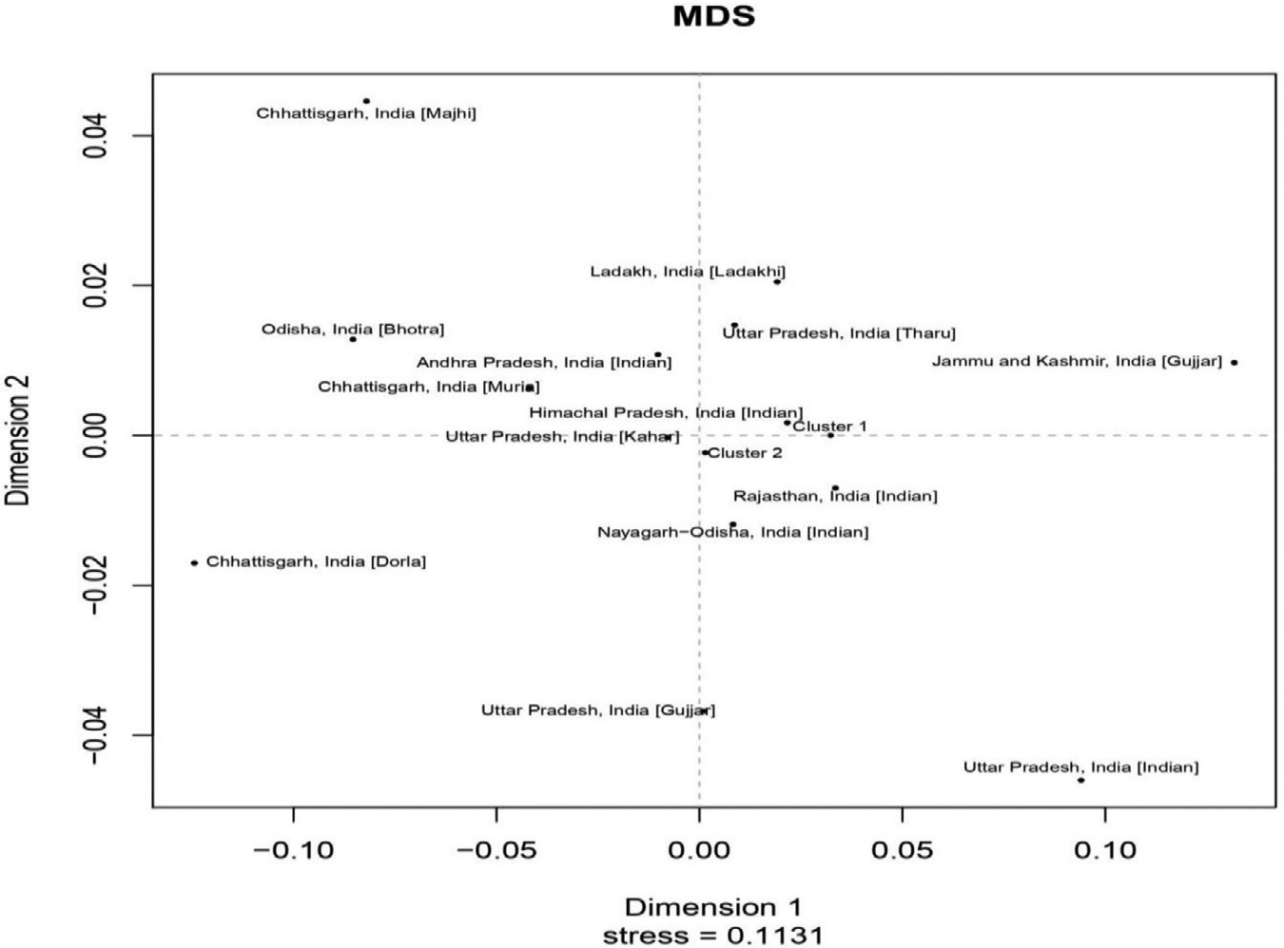
MDS plot showing the relationship of Rajasthan Brahmin population with other reference Indian populations on 23 Y-STR loci using pairwise genetic distance (R_st_) and Analysis of molecular variance (AMOVA).

Gene diversity (GD) for the present population varies from 0.438 at DYS391 locus to 0.801 at DYS576 locus. All the examined loci were found with an average gene diversity value of 0.638 with a standard error (SE) value of ± 0.025 (Supplementary Table 3). Haplotype Diversity (HD) and Discrimination Capacity (DC) values for 23 Y-STR loci were 0.645 and 0.9894, respectively. Forensic parameters including Gene Diversity (GD), Polymorphic Information Content (PIC), Probability of Match (PM) and Power of Discrimination (PD) are given in Supplementary Table 4. Different numbers of micro-variant alleles were observed at specific loci. One micro-variant allele 17.1 was found at DYS576, 24.3 at DYS390, while the four micro-variant alleles (22.1, 23.1, 24.1 and 25.1) were found at the DYS481 locus. Micro-variant allele 24.1 was found in four individuals followed by 25.1 in three individuals. The presence of these micro-variant alleles was also confirmed by repeating the amplification, capillary electrophoresis and genotyping process. The DC and GD values of the present population were almost similar to the previously reported data on the Rajasthan population. High values of DC, HD and GD show a broad scope of 23 Y-STRs in the Brahmin population of Rajasthan. To examine the genetic relatedness, the present Brahmin population was compared with other reported populations of India available at YHRD and are envisioned in Multidimensional scaling (MDS) plot (Fig. 2).

**Fig. 3:**
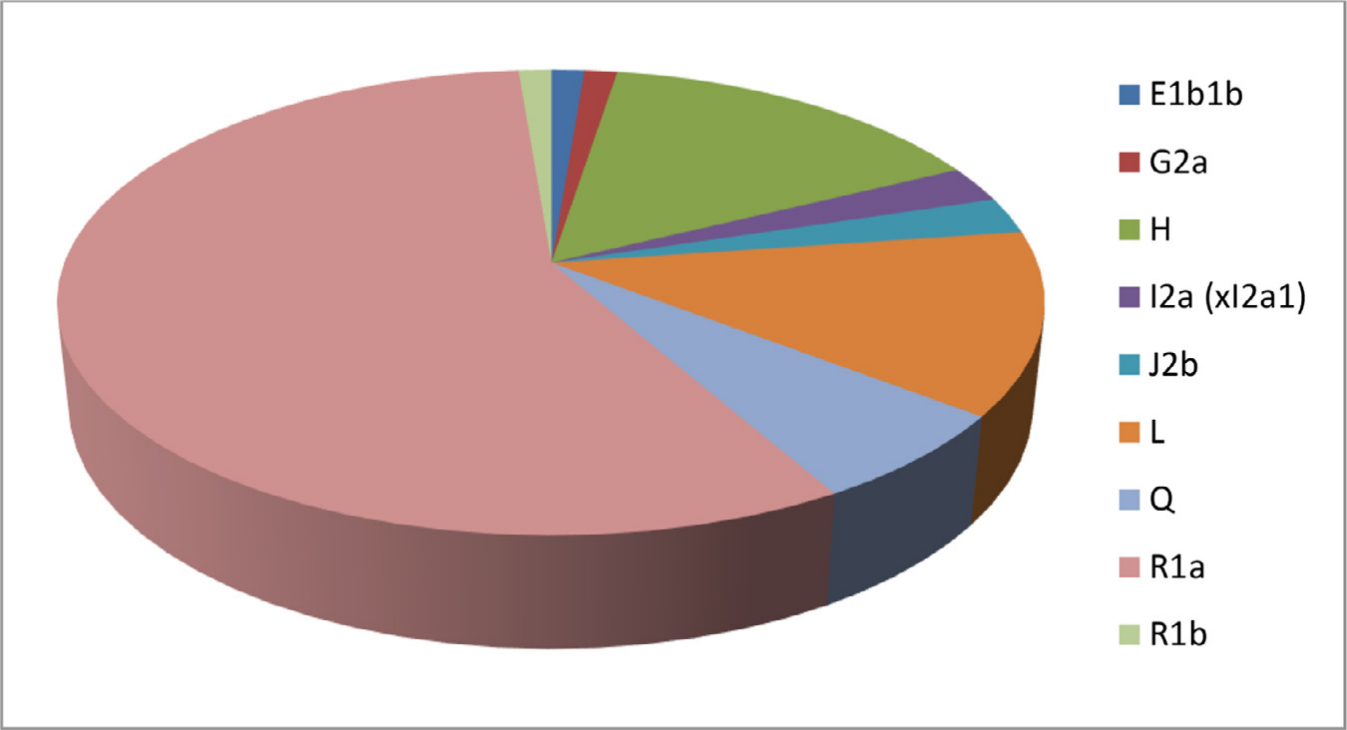
Percentage frequency of Y-Chromosomal haplogroups in Rajasthan Brahmin population assigned by Whit-Athey's algorithm.

The pairwise genetic distance (R_st_) between the present population and reference populations from the YHRD are given in Supplementary Table 5 [1], [2], [3], [4], [5], [6], [7]. The YHRD accession numbers of reference populations are listed in Supplementary Table 6. Two clusters of related Indian populations were obtained with reference populations and Brahmin population of Rajasthan. The present population lies in Cluster 1 along with Haryana, India [Brahmin] and Maharashtra, India [Indian]. Cluster 2 represents Madhya Pradesh, India [Indian]; Madhya Pradesh, India [Bhil]; Uttarakhand, India [Indian] populations. Other reference populations were observed to be genetically distant from the present population. The states Punjab, Haryana and Himachal Pradesh are geographically close and their relatedness was further confirmed by the genetic affinity of their populations. On the contrary, other geographically close populations like Uttar Pradesh, Uttarakhand, Ladakh and Jammu & Kashmir are genetically distant. The genetic relatedness of presently analysed population was observed with North Indian populations, whereas Chhattisgarh, India [Dorla] was allocated in completely different coordinates both geographically and genetically. Using Whit-Athey's algorithm [8], ten haplogroups (E1b1b, G2a, H, I2a (xI2a1), J1, J2b, L, Q, R1a and R1b) were obtained for Rajasthan Brahmin population from which R1a (56.96%), H (15.18%) and L (12.65%) were the most significant haplogroups (Fig. 3). Highest obtained frequency of R1a haplogroup is in accordance with the previous data reported from the Rajasthan population [9].

## 2 Experimental Design, Materials and Methods

### 2.1 Ethics statements

Written consent was obtained from all the participants. Ethical clearance was approved from the Institutional Human Ethical Committee (IHEC) of Maharshi Dayanand University, Rohtak, Haryana, India vide letter no. IHEC/2017/110 dt. 04/07/2017.

### 2.2 Sample collection

Blood samples of 100 unrelated healthy male volunteers from the Brahmin population of Rajasthan, India were collected on FTA cards. All the relevant information including native place, gotras/clan and geographical distribution or migration history of the participants and their ancestors was collected with the help of questionnaire. People having the family history of inter-caste marriage in the last three generations has been excluded.

### 2.3 DNA isolation, amplification and STR genotyping

DNA extraction from 100 samples was performed with FTA Card method [10]. The amplification of complete set of 23 Y-STRs was done with the help of PowerPlex®Y23 multiplex kit (Promega, USA) as per manufacturer's instructions [11]. The amplified products were size-fractionated using ABI 3130 Genetic Analyzer with the help of POP-4 polymer and the alleles were assigned by GeneMapper ID v3.2 software (Thermo Fisher Scientific, USA) [12]. WEN ILS 500 was used as an internal lane standard. All experiments fulfilled the criteria of kit controls and internal standards of the laboratory. Negative and positive controls were run concurrently with each sample batch. Generated data of 95 samples was submitted on the YHRD and accession number YC000476 was obtained for the present population data. The raw data of the population is given in Supplementary Table 7.

### 2.4 Data analysis

Singletons and frequencies of haplotypes were calculated by counting the number of unique haplotypes. Calculation of HD was done by using the formula HD = n (1- Σ pi^2^)/(n-1), in which n = number of total haplotypes in the single dataset and pi = frequency of i^th^ haplotype. Calculation of DC was done using the formula DC = h/n, in which h = the total number of unique haplotypes. Allelic frequency and HD were calculated by employing GenALEx v6.5 software [13]. Other forensic parameters including PIC, PM and PD were calculated with the help of STRAF software [14]. Analysis of molecular variance (AMOVA) and MDS plot were performed by using the tools available on YHRD website [15].

### 2.5 How to compare the data in YHRD

The generated Y-STR data can be compared as described below.

1. Visit the website https://yhrd.org.
2. Click on tools menu and select AMOVA & MDS.
3. A registration will be required to access the tools. To register for such tools, contact at amova-registration@yhrd.org and send a brief outline of user's project and some information about the user.
4. Then one can select the populations to be compared in a pull down menu.

## CRediT Author Statement

**Ritu Yadav** and **Shivkant Sharma** conceived and designed the experiments. **Shivkant Sharma** and **Ankush Patial** performed all the wet lab experiments. **Vivek Sahajpal** and **Mugdha Singh** analyzed the data. **Ritu Yadav, Shivkant Sharma** and **Lokesh Kadian** wrote the manuscript. **Ritu Yadav, Chetna Yadav, Nisha Devi, Shalu Ranga** and **Parul Ahuja** read and improved the manuscript. All the authors participated in the discussion and provided inputs to improve the manuscript's content. All authors have read and approved the final manuscript.

## Declaration of Competing Interest

The authors declare that they have no known competing interests or personal relationships that could have appeared to influence the work reported in this paper.

## Data Availability

YC000476 (Original data) (YHRD).

## Appendix Supplementary materials

Image, application 1

## Footnotes

Supplementary material associated with this article can be found in the online version at doi:10.1016/j.dib.2022.108061.

## References

[1] Singh M., Sarkar A., Kumar D., Nandineni M.R. (2020). The genetic affinities of Gujjar and Ladakhi populations of India. Sci. Rep..

[2] Dev K., Kesharwani L., Kushwaha P., Kumar A., Srivastav K.V.V., Bhasney V., Kumawat R.K., Dixit S., Srivastava A., Chaubey G., Shrivastava P. (2021). Molecular characterization of 23 Y chromosomal STR markers in the Gurjar population of National Capital Region (NCR), India. Int. J. Legal Med..

[3] Singh M., Sarkar A., Nandineni M.R. (2018). A comprehensive portrait of Y-STR diversity of Indian populations and comparison with 129 worldwide populations. Sci. Rep..

[4] Mohapatra B.K., Chauhan K., Shrivastava P., Sharma A., Dagar S., Kaitholia K. (2019). Haplotype data for 17 Y-STR loci in the population of Himachal Pradesh, India. Int. J. Legal Med..

[5] Shrivastava P., Jain T., Trivedi V.B. (2017). Haplotype data for 17 Y-STR loci in the population of Madhya Pradesh, India. Forensic Sci. Int..

[6] Perez-Benedico D., Chennakrishnaiah S., Gayden T., Rowold D.J., Garcia-Bertrand R., Herrera R.J. (2016). Y-STR markers from Ladakh in the Himalayas. Legal Med..

[7] Jain T., Shrivastava P., Trivedi V.B. (2017). Genetic portrait of Majhi tribe of Chhattisgarh, India based on 15 autosomal STRs and 23 Y-STRs. Int. J. Legal Med..

[8] Athey T.W. (2006). Haplogroup Prediction from Y-STR values using a Bayesian-allele frequency approach. J. Genetic Geneal..

[9] Kumawat R.K., Shrivastava P., Shrivastava D., Mathur G.K. (2020). Molecular diversity of 23 Y-STR genetic markers in the population of Rajasthan, India. Meta Gene.

[10] Sahajpal V., Rajput S., Sharma T., Sharma A., Thakar M.K. (2019). Development and evaluation of a novel DNA purification buffer and protocol for blood samples on FTA cards. Forensic Sci. Int..

[11] Gao T., Yun L., Gu Y., He W., Wang Z., Hou Y. (2015). Phylogenetic analysis and forensic characteristics of 12 populations using 23 Y-STR loci. Forensic Sci. Int..

[12] Bini C., Sarno S., Tangorra E., Iuvaro A., De Fanti S., Tseghereda Y.G., Pelotti S., Luiselli D. (2021). Haplotype data and forensic evaluation of 23 Y-STR and 12 X-STR loci in eight ethnic groups from Eritrea. Int. J. Legal Med..

[13] Peakall R., Smouse P.E. (2012). GenAlEx 6.5: genetic analysis in Excel. Population genetic software for teaching and research–an update. Bioinformatics.

[14] Gouy A., Zieger M. (2017). STRAF—a convenient online tool for STR data evaluation in forensic genetics. Forensic Sci. Int..

[15] http://www.yhrd.org.

